# First person – Soma Tokunaga

**DOI:** 10.1242/bio.059398

**Published:** 2022-06-10

## Abstract

First Person is a series of interviews with the first authors of a selection of papers published in Biology Open, helping early-career researchers promote themselves alongside their papers. Soma Tokunaga is first author on ‘ Factors affecting gestation periods in elasmobranch fishes’, published in BiO. Soma conducted the research described in this article while a bachelor's student in Yuuki Kawabata's lab at the Faculty of Fisheries, Nagasaki University, Bunkyo, Nagasaki, Japan. He is now a PhD student in the lab of Yuuki Y. Watanabe at the Department of Polar Science, The Graduate University for Advanced Studies, SOKENDAI, Tachikawa, Tokyo, Japan, investigating physiological and behavioral ecology of sharks.


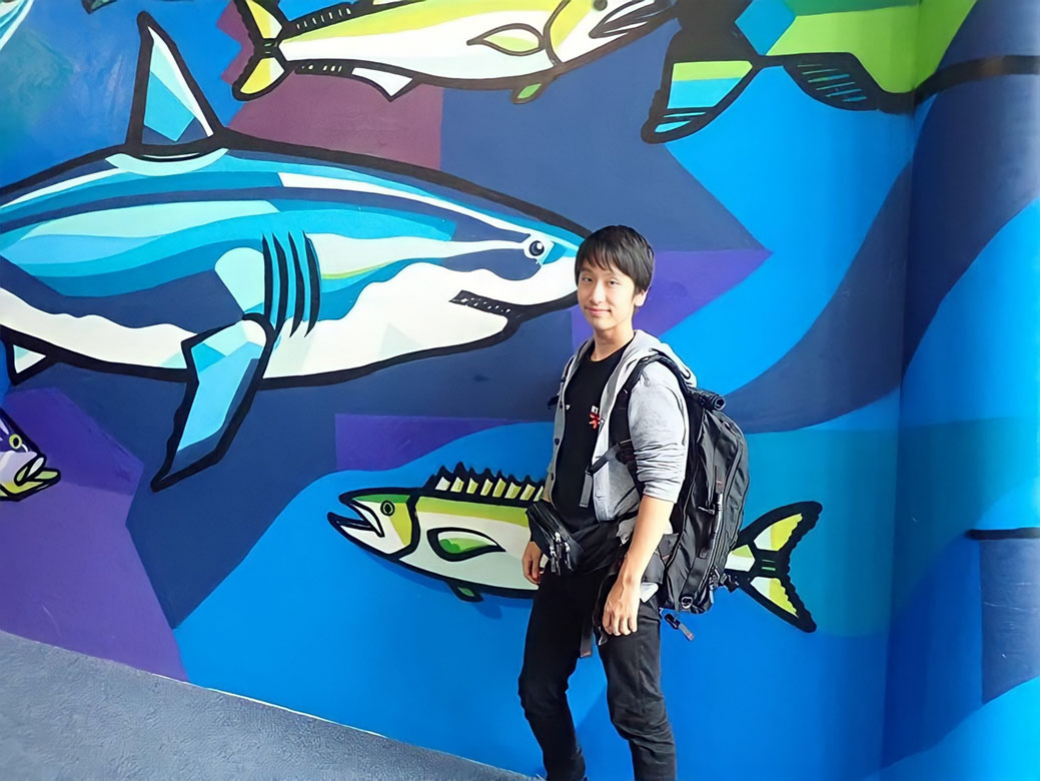


**Soma Tokunaga**

**What is your scientific background and the general focus of your lab?**

I finished my bachelor's degree from Nagasaki University in March 2021, with my senior thesis conducted in Dr Yuuki Kawabata's lab. His lab mainly studies the behaviour, ecology, and functional morphology of animals (e.g. fishes, crustaceans, insects, frogs). I then entered SOKENDAI and continued my research as a PhD student with Dr Yuuki Watanabe, studying behavioral ecology of various marine animals (e.g. sharks, penguins, seals) by using biologging technology.

**How would you explain the main findings of your paper to non-scientific family and friends?**

Gestation periods, or the duration of pregnancies, are a crucial feature of animals' reproduction. Many viviparous (producing living young) species are found in sharks and rays, and their gestation periods vary greatly among species. To identify factors causing this variation, we compiled data on gestation periods and the potential determinants from the literature. Our analyses showed that larger species tended to have longer gestation periods. A metabolism framework can explain this result: larger species have a lower metabolic rate, the rate at which organisms produce energy, for a given mass. In addition body size, body temperature and litter size (the number of offspring produced at one parturition) also affected gestation periods.

**Figure:**
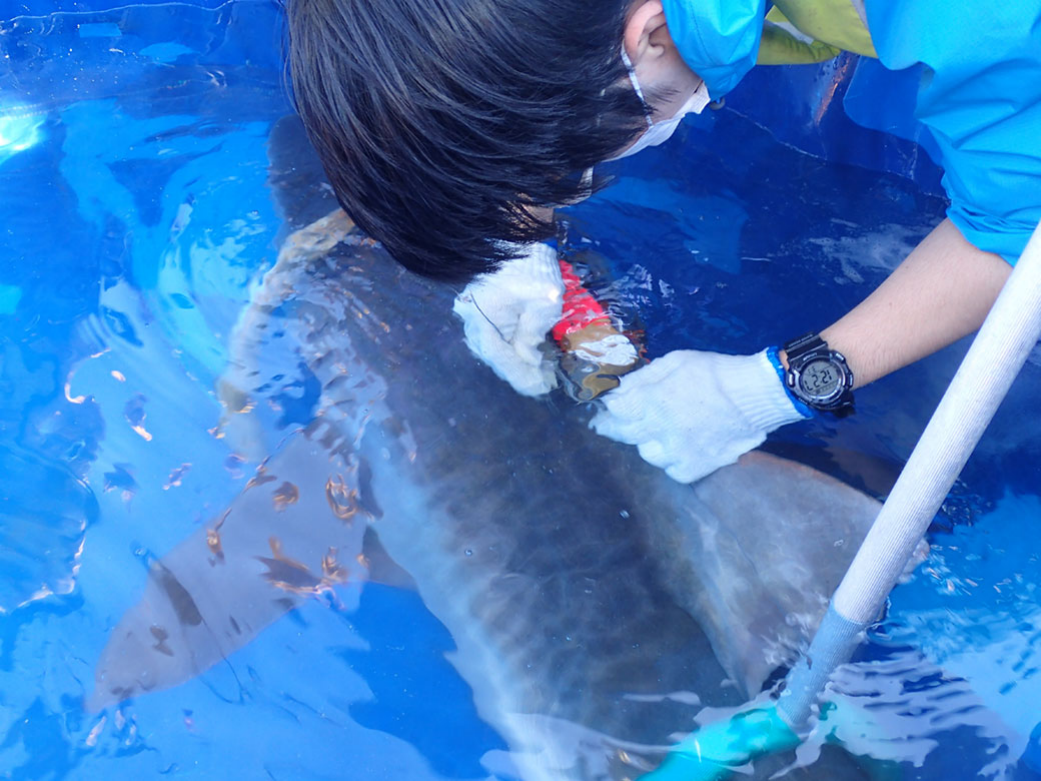
First fieldwork trip in Okinawa, Japan. Here we attached a data logger to a tiger shark.

**What are the potential implications of these results for your field of research?**

Our study provides a new perspective for understanding the life history of elasmobranch fishes. The empirical relationships shown in our study may help estimate gestation periods in species where there is little information on reproduction available. Combining our findings with intraspecific variations will be needed to understand the determinants of their gestation periods.

“The empirical relationships shown in our study may help estimate gestation periods in species where there is little information on reproduction available.”

**What has surprised you the most while conducting your research?**

We found it surprising that regionally endothermic sharks, which keep their body warmer than the ambient water around them and have elevated metabolic rates, did not show shorter gestation periods than other species. This unexpected result could be a key to understanding underlying factors determining elasmobranch gestation periods.

**What's next for you?**

As a PhD student, I started research on the behavioral ecology of sharks using biologging technology. My immediate goal is to measure the heart rate of free-swimming sharks and gain new insights into their energetics.
